# Cool and Shady: Ecophysiological Preferences of Chrysophytes

**DOI:** 10.1111/jeu.70071

**Published:** 2026-03-06

**Authors:** Christina Bock, Guido Sieber, Sara Beszteri, Frida Klein, Hannah‐Marie Stappert, Célina Wessel, Simone Engelskirchen, Jens Boenigk

**Affiliations:** ^1^ Biodiversity, Faculty of Biology University of Duisburg‐Essen Essen Germany; ^2^ Centre for Water and Environmental Research University of Duisburg‐Essen Essen Germany; ^3^ Central Collection of Algal Cultures (CCAC) University of Duisburg‐Essen Essen Germany; ^4^ Aquatic Ecology, Faculty of Biology University of Duisburg‐Essen Essen Germany

**Keywords:** alpine, Chrysophyceae, growth rates, lakes, light intensity, mixotrophy, protist, temperature

## Abstract

Chrysophyceae (Stramenopiles) are a diverse group of protists widely distributed in various aquatic habitats, including alpine lakes, where they play key ecological roles. Their nutritional modes—phototrophy, heterotrophy, and mixotrophy—enable them to adapt to the unique light and temperature conditions found across altitudinal gradients in mountain environments. This study investigates the growth responses of three mixotrophic and three phototrophic chrysophyte strains, isolated from alpine and pre‐alpine lakes, to varying light intensities and temperatures. Our results show that both temperature and light intensity exert strong, species‐specific effects on the growth of phototrophic and mixotrophic Chrysophyceae. Despite their contrasting original habitats, most strains shared similar growth optima, with peak performance generally observed between 15°C and 19°C and reduced growth at both lower and higher temperatures. All strains exhibited negative growth in darkness, confirming their phototrophic dependence. Growth rates typically increased with light availability up to a species‐specific optimum (12–35 μE m^−2^ s^−1^), beyond which either a plateau or a decline was observed. Despite environmental differences among their habitats of origin, temperature and light optima were remarkably consistent across strains.

## 1 Introduction

Chrysophyceae (Stramenopiles) are a diverse group of unicellular or colony‐forming protists occurring in a wide variety of aquatic and terrestrial habitats. They exhibit a diverse range of morphological forms, including amoeboid, palmoid, scaled, and naked cells, as well as those enclosed in a lorica or silicate scales. Their shapes can vary from spherical, oval, capsoid to filamentous forms (Scoble and Cavalier‐Smith 2014; Grossmann, Bock, et al. 2016; Kristiansen and Škaloud 2016; Malavasi et al. 2024). Chrysophytes also exhibit a notable diversity in their nutritional strategies, which allows them to thrive in various environmental conditions. Whereas many species, particularly within the Synurales (e.g., *Synura*, *Mallomonas*), are phototrophic and rely primarily on photosynthesis for carbon fixation, others are predominantly heterotrophic (e.g., *Spumella*) and therefore not dependent on light availability. However, also mixotrophy, the ability to combine both phototrophic and heterotrophic feeding modes, is widespread among chrysophytes (Mitra et al. 2016; Kristiansen and Škaloud 2016; Andersen et al. 2017; Bock et al. 2022). Whereas some mixotrophic chrysophytes are mainly phototrophic, supplementing their energy intake through bacterivory (Caron et al. 1993; Jones and Rees 1994; Rottberger 2013; Pietsch and Arndt 2024), others species use phototrophy only as a supplementary carbon uptake route and belong to the important bacterivores in numerous ecosystems (Pålsson and Daniel 2004; Boëchat et al. 2007).

Chrysophytes are especially abundant and diverse in freshwater ecosystems, thriving across a broad range of lake types, including oligotrophic, mesotrophic, and eutrophic systems (Tolotti et al. 2003; Remias et al. 2013; Bock et al. 2014, 2022; Grossmann, Jensen, et al. 2016; Khomich et al. 2017; Schagerl et al. 2025). A study on Chrysophyceae from 218 freshwater lakes in Europe highlighted the prevalence of this group as one of the most common ones in freshwater ecosystems, with Chrysophyceae detected in 213 sites and frequently ranking among the most dominant taxa (Bock et al. 2022). However, their presence is not limited to freshwater habitats; certain species and groups are also found in soil, brackish waters, and even marine environments (Findenig et al. 2010; Singer et al. 2021; Zhang et al. 2022; Zhang and Lv 2022).

Temperature and light intensity are key environmental factors that influence the distribution, growth of taxa and community composition (Sommer et al. 2012; K. Hancke et al. 2014; Wirth et al. 2019; Bock et al. 2020; Filiz et al. 2020; Lengyel et al. 2023). Temperature affects enzymatic activity, cellular respiration, photosynthesis and overall energy balance, thereby influencing growth rates, reproduction, and survival. Light availability, alone or in combination with temperature fluctuations, directly affects photosynthesis, impacting energy production and biomass accumulation (Bialevich et al. 2022; Giossi et al. 2025). Chrysophytes are particularly adapted to low light environments, often flourishing during winter or early spring when light is limited (Rott 1988; Bock et al. 2014; Öterler 2017; Schagerl et al. 2025). Their pigment composition and the ability to form photoprotective pigments enable them to survive under varying light intensities (Tanabe et al. 2011; Boenigk et al. 2023; Škaloud et al. 2023).

Light and temperature considerably differ between different habitats, in particular along altitudinal gradients. Further, protists may be differently adapted to light and temperature depending on their habitat of origin: Mid‐European lowland lakes are not or only briefly ice‐covered and reach surface water temperatures of close to 30°C in summer. In contrast, high alpine lakes are ice‐covered for several months and surface water temperatures stay much cooler. With respect to light conditions, high alpine lakes are exposed to comparatively high UV radiation and are usually characterized by a low turbidity. In contrast, lowland lakes usually have a higher turbidity and receive less UV irradiation. Thus, temperature and light regimes may differ considerably between high‐mountain lakes and pre‐alpine lakes.

To account for potential adaptations of chrysophytes to alpine habitats, we isolated strains from lakes along an altitudinal gradient ranging from 500 m a.s.l. in the pre‐alpine region to 1700 m a.s.l. in the central Alps, with the aim of examining the ecological and physiological adaptations of representative chrysophyte species. We investigated the effects of light intensity and temperature on the growth performance of six chrysophyte strains, comprising three mixotrophic and three phototrophic strains. We postulated that (1) pre‐alpine and alpine strains are in general adapted to low temperatures and high light levels (2) increasing temperature or light levels will affect chrysophytes of different nutrition modes in dissimilar ways. On the one hand we expected higher light tolerance in case of photosynthetic strains, whereas increased temperature was expected to be beneficial for mixotroph ones. To test these hypotheses, we conducted growth experiments under varying light and temperature conditions in batch cultures.

## 2 Material and Methods

### 2.1 Sampling Sites

**FIGURE 1:**
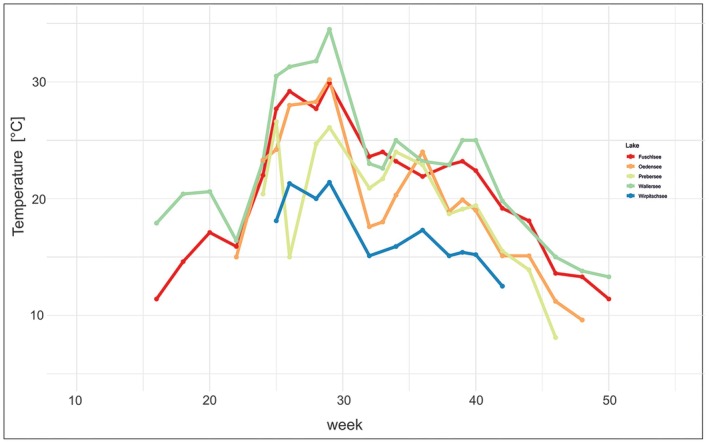
Seasonal temperature trends for individual lakes during the ice‐free period. Calendar week is shown on the x‐axis.

Four lakes within a transect from the pre‐Alps to the central Alps were sampled in 2006 and chrysophyte strains were isolated from these lakes. The transects reached from the foothills of the Alps molasse basin via the Northern Limestone Alpes to the Crysalline of the central Alpes. Specifically, lake Wallersee (506 m a.s.l.), Fuschlsee (663 m a.s.l.), Wirpitschsee (1699 m a.s.l.), Prebersee (1514 m a.s.l.), as well as Lake Ödensee (776 m a.s.l.) were sampled (Amt der Steiermärkischen Landesregierung 2008; Land Salzburg 2025a, 2025b). Their annual temperature trends during the ice‐free season are shown in Figure 1.

### 2.2 Test Organisms and Culture Conditions

We used three mixotrophic and three phototrophic chrysophyte species in this study, isolated from one of the above‐mentioned lakes (for details, see Table 1). All xenic cultures were maintained in the culture collection of the Department of Biodiversity at the University of Duisburg‐Essen and later deposited at the CCAC culture collection. The cultures were grown in DY‐V medium (pH 8) (R. A. Andersen 2009) in a climate chamber at 15°C and a 14:10 h day: night light cycle, with a light intensity of 100 μmol photons m^−2^ s^−1^. *Limnohabitans planktonicus* (strain II‐D5) was used as the feeding bacterium for the mixotrophic strains. *Limnohabitans planktonicus* was cultivated in an inorganic basal medium (NSY medium) supplemented with 3 g L^−1^ of each of the following components: nutrient broth, soytone, and yeast extract (Hahn et al. 2003). The bacterial cultures were maintained at room temperature under continuous shaking (approx. 70 rpm).

**TABLE 1:** Details of isolated strains.

Strain designation	Strain origin/GPS‐coordinates	Date of isolation	Trophic mode
*Dinobryon sociale* OE 22 K‐D	Lake Ödensee, Austria 47°33′50.187″ N 13°49′17.253″ E	May 2006	Mixotrophic
*Kephyrion* sp. FU 36 K‐N	Lake Fuschlsee, Austria 47°48′10.919″ N 13°16′33.085″ E	September 2006	Mixotrophic
*Uroglenopsis* sp. CCAC 2977B (WA 34 K‐E)	Lake Wallersee, Austria 47° 55′ 6.053″ N 13° 11′ 31.117″ E	August 2006	Mixotrophic
*Mallomonas caudata* PR26K‐H	Lake Wallersee, Austria 47°55′6.053″ N 13°11′31.117″ E	June 2006	Phototrophic
*Mallomonas annulata* WA18K‐M	Lake Wallersee, Austria 47°55′6.053″ N 13°11′31.117″ E	May 2006	Phototrophic
*Mallomonas* sp. WI26K‐B	Lake Wirpitschsee, Austria 47°14′5.56″ N 13°36′35.75″ E	June 2006	Phototrophic

### 2.3 Experimental Setup

#### 2.3.1 Growth Rates at Different Temperatures and Light Intensities

Two sets of batch culture experiments were performed over the period of 8 days (0–7) each. Algal strains were cultivated in glass/Erlenmeyer flasks in three replicates in an end volume of 75 mL.

The first set of experiments investigated seven distinct temperature conditions (5°C, 8°C, 11°C, 15°C, 19°C, 23°C, 27°C) under a light intensity of 100 μmol m^−2^ s^−1^ with a 14:10 h day: night cycle. Due to limited availability of light cabinets, the experiment was conducted in two parts. The first part included the temperature conditions 15°C, 19°C, 23°C, and 27°C, while the second part included 15°C, 11°C, 8°C, and 5°C. The 15°C treatment was performed in both parts to serve as a reference point for comparison. Before the experiment commenced, stock cultures of all strains were acclimated to the experimental conditions for 24 h. The starting cell abundance was set at 5000 cells ml^−1^ for chrysophytes, with 20 × 10^6^ bacterial cells ml^−1^ (transferred to corresponding chrysophyte media bevor the experiment) for mixotrophic strains (Table 1). Due to their larger cell size, all *Mallomonas caudata* treatments were adjusted to 200 cells ml^−1^. For *Uroglenopsis* sp., the 27°C treatment began with 500 flagellate cells ml^−1^ due to significant cell loss suffered during the initial 24‐h temperature acclimation period. Variations in initial cell concentrations were considered negligible, as growth rates are independent of cell count, provided the detection limit is not exceeded. No nutrient limitations were detected in the experimental flasks during the experiment period. Raw data from the experiment of the phototrophic strains at the different temperatures were extracted from the Bachelor thesis of Wu (2017).

The second set of experiments focused on light intensity. The experiments were conducted at 15°C with a light: dark cycle 14:10 h in two parts: part one comprised 0 μmol m^−2^ s^−1^, 12 μmol m^−2^ s^−1^; 24 μmol m^−2^ s^−1^; 35 μmol m^−2^ s^−1^; part two comprised 175 μmol m^−2^ s^−1^; 140 μmol m^−2^ s^−1^; 100 μmol m^−2^ s^−1^; 70 μmol m^−2^ s^−1^; 35 μmol m^−2^ s^−1^. 35 μmol m^−2^ s^−1^ treatment was performed in both parts to serve as a reference point for comparison. All other settings were chosen as described above.

Subsamples were taken each day and 1 mL of fixated (Lugol solution) chrysophytes samples were counted in a 1000 μL Sedgewick‐Rafter‐counting chamber under an inverted microscope (Nikon Eclipse TS‐100, Japan) with a 100× or 200× magnification.

Growth rates were calculated from the exponential growth phase by regression analysis. Growth rates were calculated using linear regression based on the natural logarithm of cell abundance over time. The exponential growth phase was identified, and a regression line was fitted to the corresponding data points. The slope of this regression line was used to determine the specific growth rate (μ) in units of day^−1^.

Statistical analysis was performed in R (v4.2) (R Core Team 2022). The normality of the growth rates was assessed using the Shapiro–Wilk test, which indicated non‐normal distributions. Consequently, Welch's ANOVA was conducted to evaluate differences in growth rates across light intensity levels and temperatures for each strain. Post hoc comparisons were performed using Tukey's HSD test (stats package v4.4.2) (R Core Team 2024).

### 2.4 Use of AI‐Assisted Language Editing

ChatGPT was used to assist with language editing, specifically to improve grammar and readability. The tool was not used to generate scientific content. After using ChatGPT, the text was carefully reviewed, verified, and revised by the authors.

## 3 Results

**FIGURE 2:**
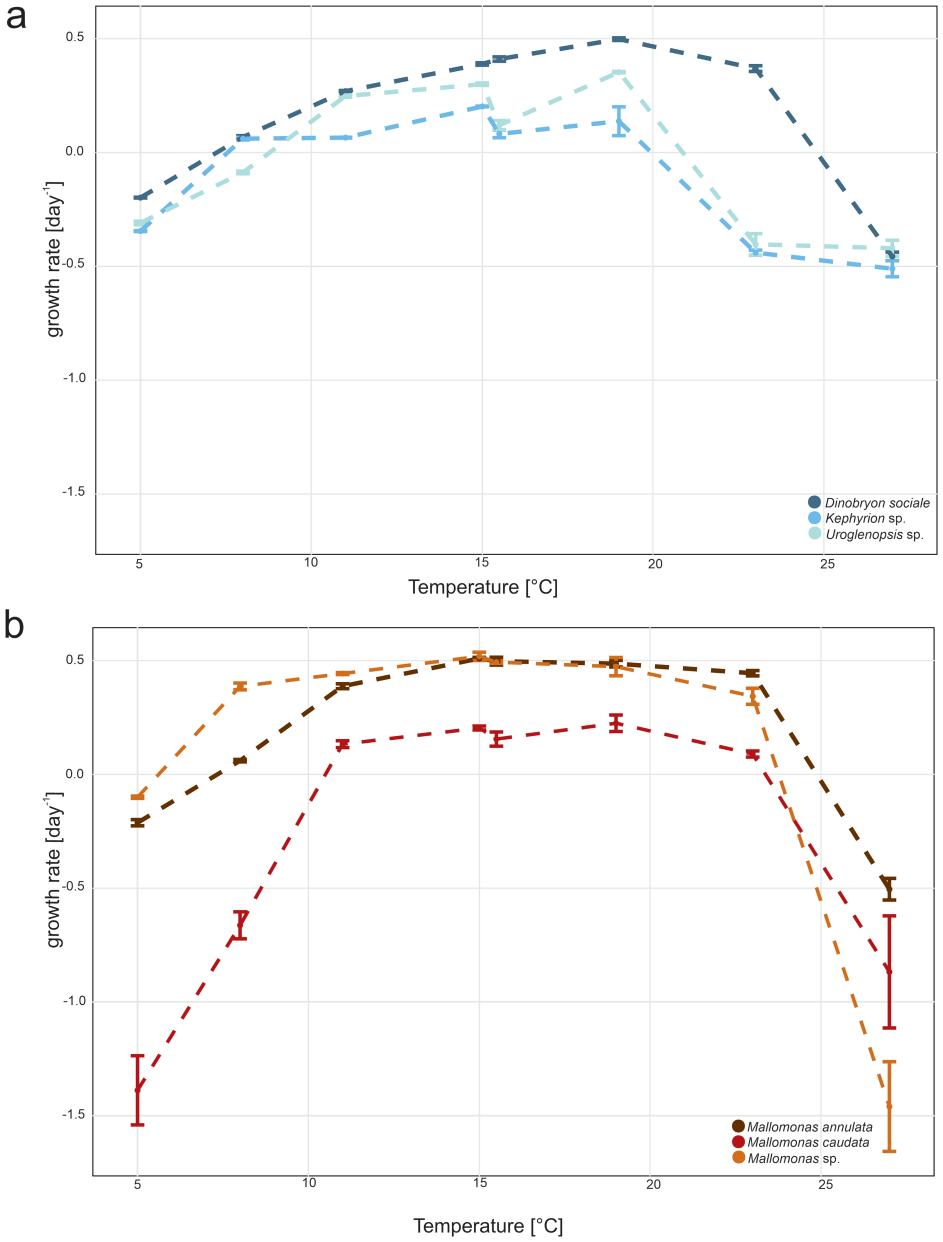
Mean growth rates (±standard deviation) of chrysophyte strains at different temperatures; (a) mixotrophic strains, (b) phototrophic strains. 15°C treatment was conducted in two independent experiments and is shown side by side for comparison.

**FIGURE 3:**
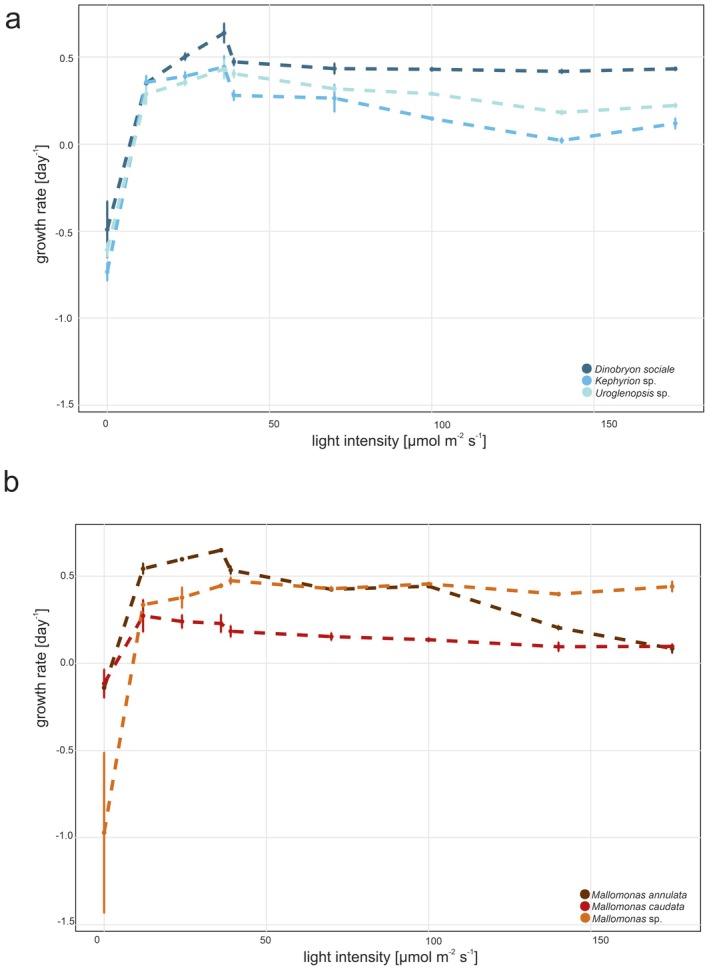
Mean growth rates (±standard deviation) of chrysophyte strains under different light intensities; (a) mixotrophic strains, (b) phototrophic strains. The 35 μE m^−2^ s^−1^ treatment was conducted in two independent experiments and is shown side by side for comparison.

The analysis of growth rates across different species and light intensities revealed distinct trends (Figures 2 and 3; Tables 2 and 3). For results of the detailed statistical comparisons, see Tables S1 and S2.

**TABLE 2:** Mean growth rates [day^1^] (with standard deviation) at different temperatures [°C].

strain	5	8	11	15	15/2	19	23	27
*Dinobryon sociale*	−0.198 (±0.002)	0.068 (±0.006)	0.270 (±0.003)	0.389 (±0.005)	0.411 (±0.010)	0.498 (±0.006)	0.369 (±0.013)	−0.456 (±0.019)
*Kephyrion* sp.	−0.345 (±0.002)	0.061 (±0.006)	0.066 (±0.002)	0.203 (±0.002)	0.082 (±0.017)	0.138 (±0.063)	−0.440 (±0.011)	−0.510 (±0.035)
*Uroglenopsis* sp.	−0.309 (±0.008)	−0.087 (±0.006)	0.248 (±0.005)	0.300 (±0.005)	0.120 (±0.020)	0.353 (±0.003)	−0.403 (±0.047)	−0.420 (±0.035)
*Mallomonas annulata*	−0.213 (±0.014)	0.059 (±0.005)	0.386 (±0.011)	0.508 (±0.005)	0.496 (±0.017)	0.485 (±0.015)	0.443 (±0.012)	−0.505 (±0.048)
*Mallomonas caudata*	−1.389 (±0.152)	−0.664 (±0.059)	0.132 (±0.015)	0.203 (±0.009)	0.154 (±0.031)	0.224 (±0.036)	0.088 (±0.013)	−0.869 (±0.246)
*Mallomonas* sp.	−0.101 (±0.005)	0.385 (±0.015)	0.442 (±0.004)	0.517 (±0.018)	0.492 (±0.006)	0.472 (±0.040)	0.342 (±0.035)	−1.460 (±0.197)

**TABLE 3:** Mean growth rates [day^1^] (with standard deviation) under different light intensities [μmol m^−2^ s^−1^].

Strain	0	12	24	35	35/2	70	100	140	175
*Dinobryon sociale*	−0.491 (±0.163)	0.350 (±0.012)	0.502 (±0.021)	0.472 (±0.021)	0.638 (±0.057)	0.433 (±0.031)	0.430 (±0.010)	0.417 (±0.011)	0.432 (±0.007)
*Kephyrion* sp.	−0.734 (±0.052)	0.355 (±0.040)	0.390 (±0.027)	0.280 (±0.030)	0.443 (±0.065)	0.264 (±0.079)	0.147 (±0.001)	0.020 (±0.016)	0.118 (±0.031)
*Uroglenopsis* sp.	−0.608 (±0.042)	0.288 (±0.060)	0.355 (±0.018)	0.404 (±0.024)	0.431 (±0.056)	0.317 (±0.016)	0.289 (±0.004)	0.181 (±0.013)	0.222 (±0.012)
*Mallomonas annulata*	−0.140 (±0.029)	0.544 (±0.032)	0.599 (±0.006)	0.536 (±0.024)	0.650 (±0.005)	0.425 (±0.005)	0.443 (±0.001)	0.205 (±0.011)	0.086 (±0.026)
*Mallomonas caudata*	−0.116 (±0.083)	0.273 (±0.094)	0.241 (±0.039)	0.185 (±0.033)	0.229 (±0.052)	0.154 (±0.022)	0.136 (±0.012)	0.096 (±0.027)	0.098 (±0.006)
*Mallomonas* sp.	−0.972 (±0.462)	0.337 (±0.012)	0.378 (±0.061)	0.475 (±0.021)	0.446 (±0.010)	0.429 (±0.013)	0.457 (±0.006)	0.398 (±0.009)	0.442 (±0.031)

### 3.1 Temperature‐Dependent Growth Responses of Mixotrophic and Phototrophic Strains

In general, all mixotroph strains investigated (*Dinobryon, Kephyrion* and *Uroglenopsis*) showed best growth at 19°C, whereas no growth was observed at the lowest and highest temperatures tested, 5°C and 27°C, respectively.

For *Dinobryon*, the mean growth rate was initially negative at 5°C (−0.198 day^−1^) but increased significantly with rising temperatures, peaking at 19°C with the highest observed growth rate (0.498 day^−1^, *p <* 0.01). At 23°C, the growth rate was significantly lower than at 19°C (0.369 day^−1^, *p <* 0.01). At 27°C, it declined further to the lowest recorded value (−0.456 day^−1^, *p <* 0.01). No significant differences were observed between the two 15°C treatments (*p* = 0.162) (Figure 2).

A similar trend was observed in *Kephyrion*, where values increased, from being negative at 5°C, up to 19°C (0.138 day^−1^) but declined sharply at 23°C (−0.44 day^−1^, *p <* 0.01) and 27°C (−0.51 day^−1^, *p =* 0.074). Significant differences were observed between the two 15°C treatments (0.203 vs. 0.08; *p <* 0.01) (Figure 2).

For *Uroglenopsis*, initial values were negative at 5°C (−0.309 day^−1^) and 8°C (−0.087 day^−1^) with significant differences between these temperatures (*p <* 0.01). A significant increase was observed at 11°C (*p <* 0.01). In the first experiment, the growth rate at 15°C was not significantly higher than at 11°C (*p =* 0.272). However, the two 15°C treatments showed no significant differences (*p <* 0.01), with the second treatment exhibiting lower growth rates (mean 0.12 vs. 0.30 day^−1^). The highest mean growth rate was recorded at 19°C (0.353 day^−1^), which was significantly higher than the second 15°C treatment (*p* < 0.01) but not significantly different from the first 15°C treatment (*p* = 0.262). At 23°C growth rate declined significantly to −0.403 day^−1^ (*p* < 0.01) and at 27°C to −0.42 day^−1^ (*p* = 0.979) (Figure 2).

Similar to the mixotroph ones, all phototroph strains exhibited negative growth at the lowest and highest temperatures tested.

Considering *Mallomonas annulata*, negative growth could be detected at 5°C (−0.213 day^−1^) followed by a positive trend from 8°C (0.059 day^−1^, *p <* 0.01) to 11°C (0.386 day^−1^, *p <* 0.01) and to 15°C (0.508 day^−1^, *p <* 0.01) before slightly but not significantly (*p =* 0.997) decreasing at 19°C (0.485 day^−1^) and 23°C (0.443 day^−1^, *p =* 0.244). However, at 27°C, the value dropped to the lowest growth rate (−0.505 day^−1^, significant *p <* 0.01 for all treatments). No significant differences were observed between the two 15°C treatments (*p* = 0.995) (Figure 2).

In contrast, *Mallomonas caudata* exhibited more variable responses. Growth rates were negative at 5°C (−1.389 day^−1^) and 8°C (−0.664 day^−1^, *p* < 0.01) but became positive at 11°C (0.132 day^−1^, *p* < 0.01). A further increase was observed at 15°C (0.202 day^−1^, *p* = 0.989), followed by a peak (not significant 15 vs. 19°C, *p* = 0.999 and 0.990 between experiments) at 19°C (0.224 day^−1^). Growth then declined at 23°C (0.088 day^−1^, *p =* 0.765) and became negative again at 27°C (−0.869 day^−1^) (Figure 2).

*Mallomonas* sp. WI26K‐B exhibited negative growth at 5°C (−0.101 day^−1^). Growth rates increased steadily from 8°C (0.385 day^−1^, *p* < 0.01) to a higher value at 11°C (0.442 day^−1^, *p* < 0.975) where a plateau was reached with no significant differences at 15°C (0.517 day^−1^, *p =* 0.896) and 19°C (*p =* 1), followed by a slight decline at 23°C (0.342 day^−1^, *p* = 0.403). However, at 27°C, growth dropped drastically to −1.46 day^−1^ (*p* < 0.01) (Figure 2).

Overall, all species exhibited optimal growth between 15°C and 19°C, while temperatures above 23°C negatively impacted growth, with 27°C causing the most significant declines.

### 3.2 Light‐Dependent Growth Responses of Mixotrophic and Phototrophic Chrysophyte Strains

In general, all species investigated showed negative growth under no light conditions. However, all light levels resulted in positive growth, and best growth was observed at light levels lower than 50 μE independent of nutrition modes.

*Dinobryon* displayed varying responses to light intensity across the different tested light levels. It showed negative growth under constant dark conditions, with a growth rate of −0.491 day^−1^.

Starting at 12 μE, *Dinobryon* exhibited positive growth (0.35 day^−1^, *p <* 0.01), and at 24 μE, the growth rate increased to 0.502 day^−1^ (*p =* 0.102). Significant differences were observed between the two growth rates at 35 μE (0.472 day^−1^ and 0.638 day^−1^ respectively, *p =* 0.062). At 70 μE (0.433 day^−1^) was a slight decrease in growth compared to 35 μE (*p =* 0.012), and the growth rate then stabilized without any significant differences at higher light levels (Figure 3).

*Kephyrion* exhibited varying growth responses to light intensity across the different tested light levels. Under constant dark conditions, it showed negative growth with a rate of −0.734 day^−1^.

At 12 μE, *Kephyrion* demonstrated a positive growth rate (0.355 day^−1^, *p <* 0.01), but the mean growth slightly decreased at 24 μE (0.39 day^−1^, *p =* 0.976). At 35 μE, the growth rate dropped further to 0.28 day^−1^ (*p =* 0.082). A slightly higher growth was observed at the second run at 35 μE (0.443 day^−1^, *p <* 0.01). At 70 μE, the growth rate decreased to 0.264 day^−1^ (*p <* 0.01) and continued to decline at 100 μE but only significantly compared to 35 μE (0.147 day^−1^, *p <* 0.01). At 140 μE (0.02 day^−1^) and 175 μE (0.118 day^−1^), it further declined. There were no significant differences between the growth rates at these higher light levels, indicating a plateau in growth (Figure 3).

Under constant dark conditions, the growth rate of *Uroglenopsis* sp. was negative, at −0.608 day^−1^.

At 12 μE, *Uroglenopsis* exhibited a positive growth rate of 0.288 day^−1^ (*p <* 0.01), which increased slightly to 0.355 day^−1^ at 24 μE (*p =* 0.318). At 35 μE, the growth rate further increased (*p =* 0.665) to 0.404 day^−1^. At 70 μE, the growth rate decreased to 0.317 day^−1^ (*p =* 0.013) and continued to decrease at 100 μE (0.289 day^−1^, *p =* 0.974). At higher light intensities of 140 μE (0.181 day^−1^) and 175 μE (0.222 day^−1^), the growth rates were still positive but showed a noticeable decline compared to the lower light levels (Figure 3).

Under constant dark conditions, *Mallomonas caudata* exhibited a negative growth rate of −0.116 day^−1^. At 12 μE, the growth rate increased significantly to 0.273 day^−1^ (*p <* 0.01), changed slightly to 0.241 day^−1^ at 24 μE (*p =* 0.995). At 35 μE, the growth rate decreased slightly to 0.185 day^−1^ (*p =* 0.890). At the second run, the growth rate of 35 μE was higher than at the first run (0.229 day^−1^, *p =* 0.966). At higher light intensities, growth rates began to decline: at 70 μE, the growth rate dropped to 0.154 day^−1^ (*p =* 0.648), and it remained almost stable at 0.136 day^−1^ at 100 μE (*p =* 1). At 140 and 175 μE, the growth rates decreased further to 0.096 day^−1^ (*p =* 0.982) and 0.086 day^−1^ (*p =* 1).

Under constant dark conditions, *Mallomonas annulata* exhibited a negative growth rate of −0.14 day^−1^. At 12 μE, the growth rate increased significantly to 0.544 day^−1^ (*p <* 0.01), and continued to rise slightly to 0.599 day^−1^ at 24 μE (*p =* 0.052). At 35 μE, the growth rate decreased slightly to 0.536 day^−1^ (*p =* 0.052). At the second run, the growth rate of 35 μE was higher than at the first run (0.65 day^−1^, *p <* 0.01). At higher light intensities, growth rates began to decline: at 70 μE, the growth rate dropped to 0.425 day^−1^ (*p <* 0.01), and it remained almost stable at 0.443 day^−1^ at 100 μE (*p =* 0.965). At 140 μE, the growth rate decreased further to 0.205 day^−1^ (*p <* 0.01), and by 175 μE, the growth rate dropped to 0.086 day^−1^ (*p <* 0.01), indicating a significant decline in growth at the highest light levels (Figure 3).

Under constant dark conditions, *Mallomonas* sp. exhibited a negative growth rate of −0.972 day^−1^. At 12 μE, the growth rate increased significantly to 0.337 day^−1^ (*p <* 0.01), changed slightly to 0.378 day^−1^ at 24 μE (*p =* 1), and 0.475 day^−1^ at 35 μE (*p =* 0.997). At the second run, the growth rate of 35 μE was slightly lower than at the first run (0.446 day^−1^, *p =* 1). At higher light intensities, growth rates became more or less stable: 0.429 day^−1^ (*p =* 1) at 70 μE, 0.457 day^−1^ at 100 μE (*p =* 1), 0.398 day^−1^ (*p =* 1) at 140, and 0.442 day^−1^ (*p =* 1) at 175 μE (Figure 3).

## 4 Discussion

Our results demonstrate that both temperature and light intensity exert strong and species‐specific effects on the growth rates of phototrophic and mixotrophic Chrysophyceae from pre‐ and alpine environments. However, despite the very different habitats of origin, the observed optima were surprisingly similar between strains and trophic modes. Across all tested strains, optimal growth was generally observed at intermediate temperatures, particularly between 15°C and 19°C. Growth was inhibited at both lower (5°C) and higher (≥ 23°C) temperatures, with the strongest declines typically occurring at 27°C. These patterns were consistent across trophic modes, although the extent and shape of the temperature response curve varied among species.

Similarly, light intensity had a pronounced effect on growth. All species exhibited negative growth under dark conditions, confirming their phototrophic dependence regardless of mixotrophic capabilities. Growth rates increased with light availability up to a species‐specific threshold, beyond which a decline was observed in some cases or a “plateau” was reached, suggesting light saturation and in some cases possible photo‐inhibition.

### 4.1 Seasonal Niches and Temperature Preferences in Chrysophyceae

The observed temperature optima for growth (15°C–19°C) suggest that the studied taxa are best adapted to spring, early summer, or autumn conditions rather than peak summer temperatures (Figure 1). Surface water temperatures typically reached the optimal range for growth by early June, irrespective of altitude (Figure 1). While surface temperatures can occasionally exceed 27°C, integrated water samples—including temperatures from both surface and deeper cooler water zones—show consistently lower average temperatures. Notably, Bock et al. (2014) observed in a molecular seasonal study of lake Fuschlsee (Austria) the persistent dominance of an *Uroglenopsis (Uroglena) americana* OTU from June through October in integrated samples, implying a wide temporal distribution across warm seasons. However, this pattern appears inconsistent with our growth data for an *Uroglenopsis* isolate, which showed a marked decline in growth rate above 19°C. Long periods of dominance throughout the year have also been documented for other phototrophic Chrysophyceae (Bock et al. 2014). However, all the species tested in our study died at 27°C. One possible explanation lies with the thermal optimum within the water column: taxa sensitive to elevated surface temperatures may migrate into deeper, cooler layers to avoid heat stress. **V**ertical migration is already documented for different chrysophytes, allowing them to alternate between light‐rich upper layers and nutrient‐ or prey‐rich deeper zones (Jones 1988; Kim and Takamura 2001; Heinze et al. 2013). Unfortunately, vertical temperature profiles were not conducted in our study, limiting our ability to precisely localize thermal niches in the water column.

Conversely, negative growth at 5°C in our isolates likely excludes them from active participation in winter phytoplankton communities, favoring psychrotolerant taxa instead. Nevertheless, several studies have reported that certain chrysophyte taxa can attain high relative abundances during winter. In particular, members of the genera *Synura*, *Dinobryon*, *Mallomonas*, and *Chrysococcus*, have been documented in boreal, alpine, and shallow lakes, in some cases even under ice cover (Simon et al. 2015; Agbeti and Smol 1995; Rott 1988; Vehmaa and Salonen 2009; Öterler 2017; Chiapella et al. 2021; Socha et al. 2023). These findings suggest that certain chrysophyte taxa possess adaptations that enable them to thrive under cold and ice‐covered conditions and confirm that specific thermal adaptations can occur within and between closely related species (Lee and Kim 2007; Nolte et al. 2010; Bock et al. 2014).

In the context of ecological niches, compared to less‐studied genera such as *Uroglenopsis* and *Kephyrion*, the mixotrophic genus *Dinobryon* has been relatively well characterized. Previous studies indicate that its temperature‐dependent growth is highly species‐ and strain‐specific: Wirth et al. (2019) found stable growth between 10°C and 15°C, with higher temperatures combined with intense light promoting growth in certain strains. For example, a *Dinobryon sociale* strain grew above 20°C only under specific conditions, consistent with our observations of reduced growth at 23°C and no growth at 27°C. Further studies by Princiotta et al. (2016) and Heinze et al. (2013) reinforce the species‐ and strain‐specific nature of temperature responses. Similar patterns are reported for phototrophic chrysophytes like *Mallomonas*. Different Korean strains all displayed optimal growth between 18°C–21°C (Lee and Kim 2007), aligning in several parts with our results.

Bock et al. (2022) investigated the distribution of phylogenetic lineages within Chrysophyceae across European lakes using environmental data on the V9 region of the SSU. Among their findings, they showed that lineages associated with *Mallomonas* sp. WI26KB were found across temperature gradients ranging from 15°C to 19°C. Similarly, lineages linked to *Dinobryon sociale* and *Uroglenopsis* occurred across a temperature span of up to 14°C.

These observations illustrate the considerable thermal flexibility of certain chrysophyte lineages in natural environments, highlighting their potential to occur under varying climatic conditions.

Despite taxon‐ and strain‐specific variation, our study indicated nutritional type‐related patterns: Mixotrophic strains showed a more pronounced temperature optimum, while in phototrophic strains this was less pronounced. This contrast may reflect differing temperature sensitivities of autotrophic vs. heterotrophic processes, as proposed by the metabolic theory of ecology (Allen et al. 2006; López‐Urrutia et al. 2006; Rose and Caron 2007), which basically states that heterotrophic processes respond more strongly to temperature as compared to autotrophic processes. This interpretation is supported by findings on *Ochromonas*, a predominantly phagotrophic mixotroph chrysophyte, which increased its reliance on heterotrophy with rising temperatures (Wilken et al. 2014). In contrast, no such temperature‐driven shift was observed in *Dinobryon sociale* , a species primarily dependent on phototrophy (Princiotta et al. 2016). These findings highlight how trophic strategy may shape thermal response patterns in chrysophytes.

### 4.2 Light Intensity

Light intensity affects cells, particularly photosynthesis, and excessive light can lead to photooxidative damage (Long et al. 1994; Choudhury and Behera 2001). All of our six chrysophyte taxa died in darkness and showed strain‐specific responses to increasing light intensity. Optimal growth occurred at low to moderate light levels (12–35 μE). Higher intensities (≥ 70 μE) led to reduced growth in all species, with *Kephyrion* and *Mallomonas annulata* showing the strongest decline, indicating varying degrees of photoinhibition. These findings suggest species‐specific light preferences, with most taxa performing best under low to moderate light conditions.

The study by Wirth et al. (2019) showed that three examined *Dinobryon* species—*D. divergens, D. sertularia*, and *D. sociale* —exhibited reduced growth rates at low light intensities (10 μE), and this reduction became more pronounced at higher temperatures.

In our experiments, *D. sociale* reached its highest growth rate already at 36 μE, and increasing light intensity beyond this point did not lead to further enhancement of growth. This discrepancy may reflect strain‐specific differences or methodological variations between studies and strains. It also suggests that *D. sociale* may have a relatively narrow optimal light range, beyond which additional light does not yield physiological benefits, potentially due to light saturation or energy imbalances in the photosynthetic apparatus.

In a study by Kim et al. (2009), batch cultures of three strains of *Mallomonas caudata* were used to investigate, among others, the effect of light intensity on growth. The maximum growth rates occurred between 42 and 104 μmol m^−2^ s^−1^, depending on the strain and temperature. Population growth occurred over a wide range of nutrient and light intensities, but there were strain‐specific differences that, according to the authors, may represent adaptations to local environments (Kim et al. 2009). This study was partially able to demonstrate the “plateau” effect that we observed more prominently in our experiments with phototrophic species. However, higher light intensities were not tested in the study of Kim et al. (2009), which limits the ability to confirm this pattern with certainty.

Another study focused on *Mallomonas elongata* , a bloom‐forming species. *M. elongata* exhibited growth at all experimental light intensities (10–130 μmol m^−2^ s^−1^), but very low growth appeared below 20 μmol m^−2^ s^−1^. Growth rates increased with light intensity up to 50–80 μmol m^−2^ s^−1^, with temperature influencing the optimal light intensity for growth (Kim and Lee 2011). In contrast to *M. caudata* , a clear decline in growth rate with increasing light intensity was observed in *M. elongata* . A plateau—where growth remains stable despite changes in light intensity—was not observed in this case.

Chrysophyceae typically possess two flagella and are capable of active movement within the water column (Andersen 1989). This ability for directed movement enables chrysophytes to actively migrate in response to their specific preferences for light intensity and spectral quality (Fee 1976; Nygaard 1977; Pick et al. 1984; Kim and Takamura 2001). Several studies have documented diel vertical migration in chrysophytes, particularly in stratified lakes. During the daytime, many species remain in the upper water layers to perform photosynthesis. At night, they migrate to deeper, bacteria‐rich zones to feed phagotrophically (Croome and Tyler 1988; Jones 1988, 2000). We have shown that both mixotrophic and phototrophic tested chrysophytes generally prefer low light intensities, suggesting that their vertical distribution within the water column is closely tied to light availability. It is therefore plausible that species with a stronger affinity for low light conditions may occupy deeper layers of the epilimnion or even move into the metalimnion, particularly in clear, stratified lakes. Such behavior would result in a pronounced vertical structuring of Chrysophyceae populations according to light gradients. Consequently, when sampling chrysophyte communities, it is crucial to account for both light intensity and depth to avoid underrepresentation of specific taxa.

Chrysophyceae possess characteristic photosynthetic pigments, including chlorophyll *a* and *c*, as well as various accessory carotenoids, most notably fucoxanthin (Boenigk et al. 2023; Škaloud et al. 2023). Fucoxanthin and related pigments absorb light in the blue and green regions of the spectrum, which are more prevalent in deeper or low‐light aquatic layers, thereby extending the range of light usable for photosynthesis beyond that of chlorophyll alone (Gelzinis et al. 2015; Pajot et al. 2022; Khaw et al. 2022). This can allow Chrysophyceae to utilize light more efficiently under low‐light conditions, which can be advantageous in clear, stratified lakes. Experimental evidence indicates that fucoxanthin‐containing microalgae adjust their pigment composition in response to low light conditions, often increasing fucoxanthin content to enhance light harvesting under suboptimal illumination (Chinnappan et al. 2024). Differences in pigment composition and light‐harvesting strategies among algal classes drive distinct photosynthetic responses to light intensity. Variation in pigment profiles across photosynthetic lineages (e.g., chlorophyll *a/b*, chlorophyll *a/c*, fucoxanthin, and other carotenoids) underpins their ability to exploit different ecological niches (Lehmuskero et al. 2018; Maltsev et al. 2021).

While some protists are capable of surviving in a wide range of environmental conditions, their actual distribution and growth are often more limited. Fundamental niches, based on controlled laboratory experiments or environmental models, do not always align with the realized niches that protists occupy in nature. For example, species may tolerate a broader temperature range under controlled laboratory conditions than they encounter in the wild, due to multiple ecological factors that cannot be attributed to a single factor. On the other hand, microevolutionary changes during cultivation could, in principle, alter physiological responses. However, the observed ecophysiological optima of the tested strains in our study differ in several cases from the culture conditions, making such adaptation unlikely. Furthermore, our Arctic Chrysophyceae isolates (Boenigk pers. comment; data not shown) have been maintained in culture for decades without any apparent increase in temperature tolerance. They continue to exhibit a clear upper thermal limit, suggesting that their temperature responses largely reflect their original ecological adaptations rather than long‐term effects of cultivation.

Overall, our results indicate that the interplay between temperature and light availability defines distinct ecological niches and physiological optima for Chrysophyceae, with implications for their distribution and success under changing environmental conditions in pre‐ and alpine lake ecosystems. All strains showed a clear preference for low light conditions, indicating a general adaptation to low‐irradiance environments. Despite differences in habitat elevation, the temperature and light intensity optima were remarkably similar across strains, suggesting shared evolutionary constraints and selective pressures among Chrysophyceae lineages. Mixotrophic strains exhibited slightly more defined, narrower optima compared to phototrophic ones, which may reflect differing metabolic flexibility between the two nutritional modes.

## Funding

This work was supported by Deutsche Forschungsgemeinschaft, 426547801.

## Supporting information


**Table S1:** Results of the statistic significant tests for growth rates under different temperatures [°C].

**Table S2:** Results of the statistic significant tests for growth rates under different light intensities [μmol m^−2^ s^−1^].

## Data Availability

The data that support the findings of this study are available from the corresponding author upon reasonable request.
