# Supplementary material for: Cool and Shady: Ecophysiological Preferences of Chrysophytes
Source: J Eukaryot Microbiol. 2026 Mar 6;73(2):e70071. doi: 10.1111/jeu.70071 (PMC12966406; doi:10.1111/jeu.70071)
Supplement: Supplementary file 1 — Table S1: Results of the statistic significant tests for growth rates under different temperatures [°C]. [file JEU-73-e70071-s001.docx]

**Table S1:** Results of the statistic significant tests for growth rates under different temperatures [°C]

| Treatment temperature [°C] | *Dinobryon sociale* | *Kephyrion* sp*.* | *Uroglenopsis* sp*.* | *Mallomonas caudata* | *Mallomonas annulata* | *Mallomonas* sp*.* |
| --- | --- | --- | --- | --- | --- | --- |
| 5 vs 8 | <0.01 | <0.01 | <0.01 | <0.01 | <0.01 | <0.01 |
| 8 vs 11 | <0.01 | 1.000 | <0.01 | <0.01 | <0.01 | 0.975 |
| 11 vs 15 | <0.01 | <0.01 | 0.272 | 0.989 | <0.01 | 0.896 |
| 15 vs 15/2 | 0.162 | <0.01 | <0.01 | 0.999 | 0.995 | 1.000 |
| 15/2 vs 19 | <0.01 | 0.239 | <0.01 | 0.990 | 0.997 | 1.000 |
| 19 vs 23 | <0.01 | <0.01 | <0.01 | 0.765 | 0.244 | 0.403 |
| 23 vs 27 | <0.01 | 0.074 | 0.979 | <0.01 | <0.01 | <0.01 |
| 5 vs 11 | <0.01 | <0.01 | <0.01 | <0.01 | <0.01 | <0.01 |
| 5 vs 15 | <0.01 | <0.01 | <0.01 | <0.01 | <0.01 | <0.01 |
| 5 vs 15/2 | <0.01 | <0.01 | <0.01 | <0.01 | <0.01 | <0.01 |
| 5 vs 19 | <0.01 | <0.01 | <0.01 | <0.01 | <0.01 | <0.01 |
| 5 vs 23 | <0.01 | <0.01 | <0.01 | <0.01 | <0.01 | <0.01 |
| 5 vs 27 | <0.01 | <0.01 | <0.01 | <0.01 | <0.01 | <0.01 |
| 8 vs 15 | <0.01 | <0.01 | <0.01 | <0.01 | <0.01 | 0.388 |
| 8 vs 15/2 | <0.01 | 0.974 | <0.01 | <0.01 | <0.01 | 0.631 |
| 8 vs 19 | <0.01 | 0.045 | <0.01 | <0.01 | <0.01 | 0.814 |
| 8 vs 23 | <0.01 | <0.01 | <0.01 | <0.01 | <0.01 | 0.995 |
| 8 vs 27 | <0.01 | <0.01 | <0.01 | 0.320 | <0.01 | <0.01 |
| 11 vs 15/2 | <0.01 | 0.993 | <0.01 | 1.000 | <0.01 | 0.987 |
| 11 vs 19 | <0.01 | 0.066 | <0.01 | 0.956 | <0.01 | 0.999 |
| 11 vs 23 | <0.01 | <0.01 | <0.01 | 0.999 | 0.050 | 0.698 |
| 11 vs 27 | <0.01 | <0.01 | <0.01 | <0.01 | <0.01 | <0.01 |
| 15 vs 19 | <0.01 | 0.119 | 0.262 | 1.000 | 0.854 | 0.993 |
| 15 vs 23 | 0.218 | <0.01 | <0.01 | 0.878 | 0.021 | 0.125 |
| 15 vs 27 | <0.01 | <0.01 | <0.01 | <0.01 | <0.01 | <0.01 |
| 15/2 vs 23 | <0.01 | <0.01 | <0.01 | 0.993 | 0.080 | 0.252 |
| 15/2 vs 27 | <0.01 | <0.01 | <0.01 | <0.01 | <0.01 | <0.01 |
| 19 vs 27 | <0.01 | <0.01 | <0.01 | <0.01 | <0.01 | <0.01 |
