# Supplementary material for: Cool and Shady: Ecophysiological Preferences of Chrysophytes
Source: J Eukaryot Microbiol. 2026 Mar 6;73(2):e70071. doi: 10.1111/jeu.70071 (PMC12966406; doi:10.1111/jeu.70071)
Supplement: Supplementary file 2 — Table S2: Results of the statistic significant tests for growth rates under different light intensities [μmol m−2 s−1]. [file JEU-73-e70071-s002.docx]

**Table S2:** Results of the statistic significant tests for growth rates under different light intensities [µmol m^-^² s^-1^]

| Treatment light intensities [µmol m^-^² s^-1^] | *Dinobryon sociale* | *Kephyrion*  sp. | *Uroglenopsis*  sp. | *Mallomonas caudata* | *Mallomonas annulata* | *Mallomona*  sp. |
| --- | --- | --- | --- | --- | --- | --- |
| 0 vs 12 | <0.01 | <0.01 | <0.01 | <0.01 | <0.01 | <0.01 |
| 12 vs 24 | 0.102 | 0.976 | 0.318 | 0.995 | 0.052 | 1.000 |
| 24 vs 35 | 0.999 | 0.082 | 0.665 | 0.890 | 0.020 | 0.997 |
| 35 vs 35/2 | 0.062 | <0.01 | 0.982 | 0.966 | <0.01 | 1.000 |
| 35/2 vs 70 | 0.012 | <0.01 | 0.013 | 0.648 | <0.01 | 1.000 |
| 70 vs 100 | 1.000 | 0.107 | 0.974 | 1.000 | 0.956 | 1.000 |
| 100 vs 140 | 1.000 | 0.032 | 0.020 | 0.982 | <0.01 | 1.000 |
| 140 vs 175 | 1.000 | 0.153 | 0.850 | 1.000 | <0.01 | 1.000 |
| 0 vs 24 | <0.01 | <0.01 | <0.01 | <0.01 | <0.01 | <0.01 |
| 0 vs 35 | <0.01 | <0.01 | <0.01 | <0.01 | <0.01 | <0.01 |
| 0 vs 35/2 | <0.01 | <0.01 | <0.01 | <0.01 | <0.01 | <0.01 |
| 0 vs 70 | <0.01 | <0.01 | <0.01 | <0.01 | <0.01 | <0.01 |
| 0 vs 100 | <0.01 | <0.01 | <0.01 | <0.01 | <0.01 | <0.01 |
| 0 vs 140 | <0.01 | <0.01 | <0.01 | <0.01 | <0.01 | <0.01 |
| 0 vs 175 | <0.01 | <0.01 | <0.01 | <0.01 | <0.01 | <0.01 |
| 12 vs 35 | 0.287 | 0.428 | 0.011 | 0.451 | 1.000 | 0.969 |
| 12 vs 35/2 | <0.01 | 0.246 | <0.01 | 0.969 | <0.01 | 0.993 |
| 12 vs 70 | 0.731 | 0.332 | 0.974 | 0.143 | <0.01 | 0.998 |
| 12 vs 100 | 0.774 | <0.01 | 1.0 | 0.064 | <0.01 | 0.987 |
| 12 vs 140 | 0.888 | <0.01 | 0.021 | <0.01 | <0.01 | 1.000 |
| 12 vs 175 | 0.744 | <0.01 | 0.315 | <0.01 | <0.01 | 0.994 |
| 24 vs 35/2 | 0.189 | 0.798 | 0.177 | 1.000 | 0.074 | 1.000 |
| 24 vs 70 | 0.878 | 0.070 | 0.897 | 0.481 | <0.01 | 1.000 |
| 24 vs 100 | 0.845 | <0.01 | 0.330 | 0.262 | <0.01 | 0.999 |
| 24 vs 140 | 0.716 | <0.01 | <0.01 | 0.044 | <0.01 | 1.000 |
| 24 vs 175 | 0.869 | <0.01 | <0.01 | 0.049 | <0.01 | 1.000 |
| 35 vs 70 | 0.995 | 1.000 | 0.090 | 0.997 | <0.01 | 1.000 |
| 35 vs 100 | 0.992 | 0.022 | 0.011 | 0.949 | <0.01 | 1.000 |
| 35 vs 140 | 0.962 | <0.01 | <0.01 | 0.454 | <0.01 | 0.999 |
| 35 vs 175 | 0.994 | <0.01 | <0.01 | 0.483 | <0.01 | 1.000 |
| 35/2 vs 100 | 0.011 | <0.01 | <0.01 | 0.393 | <0.01 | 1.000 |
| 35/2 vs 140 | <0.01 | <0.01 | <0.01 | 0.076 | <0.01 | 1.000 |
| 35/2 vs 175 | 0.012 | <0.01 | <0.01 | 0.083 | <0.01 | 1.000 |
| 70 vs 140 | 1.000 | <0.01 | <0.01 | 0.871 | <0.01 | 1.000 |
| 70 vs 175 | 1.000 | 0.026 | 0.047 | 0.891 | <0.01 | 1.000 |
| 100 vs 175 | 1.000 | 0.994 | 0.303 | 0.987 | <0.01 | 1.000 |
